# Graphene Oxide Membranes—Synthesis, Properties, and Applications

**DOI:** 10.3390/membranes13090771

**Published:** 2023-08-31

**Authors:** Natalia Chumakova, Alexander Kokorin

**Affiliations:** 1 N.N. Semenov Federal Research Center for Chemical Physics, Russian Academy of Science, Kosygin St. 4, Moscow 119991, Russia; alex-kokorin@yandex.ru; 2 Department of Chemistry, Lomonosov Moscow State University, Leninskiye Gory, 1/3, Moscow 119991, Russia

Graphene oxide (GO) is a layered material composed of graphene planes randomly decorated by oxygen-containing groups—carbonyl, hydroxyl, epoxy, carboxyl, etc. Membranes formed from graphene oxide flakes possess selective permeability for polar liquids, hydrated ions, and gases. In 2012, it was reported in [1] that dry GO membranes are highly permeable for water but surprisingly impermeable for ethanol, propanol, acetone, and even helium. At present, the unique permeability of GO membranes is explained by their complex internal structure, in particular, the existence of abundant oxidized and unoxidized nanochannels. However, the mechanism of interaction of substances intercalated into the inter-plane space with the GO surface is still not fully understood.

At present, GO membranes are considered as promising materials for water desalination and purification. Research in this area is moving in two parallel directions—study of the fundamental mechanism of the diffusion of substances inside GO nanochannels, and tuning of the membranes’ properties. This tuning can be realized by varying the oxidative level of the GO, by varying of strategy for manufacturing of membranes, by introducing dopants, etc. This Special Issue "Graphene Oxide Membranes—Synthesis, Properties, and Applications" includes articles devoted to both the fundamental properties of GO membranes and their functional parameters.

An important characteristic of the internal structure of GO membranes is the orientational order of the oxidized graphene planes. Until recently, the ordering of the membranes was assessed only qualitatively (visually) on the basis of the cross-sections of SEM and TEM images. At present, the orientational structure of the membranes can be described quantitatively using the spin probe method. This technique is based on the analysis of the EPR spectra of a membrane containing stable radicals sorbed on the oxidized graphene planes. The sensitivity of this approach depends on the chemical structure of the probing molecules. In paper [2], three novel stable nitroxide radicals were tested as spin probes to study the GO membranes’ alignment. It was shown that the presence of a conjugated aromatic fragment containing two nitrogen atoms in the structure of a radical enhances its interaction with the oxygen-containing groups located on the surface of the oxidized graphene planes and, as a result, improves the orientation of the probing molecules in the membrane.

One of the main fundamental properties of GO membranes is their ability to sorb polar liquids of various natures. In paper [3], an accurate experimental comparison of the sorption capacity of GO membranes and the corresponding graphite oxide powders is presented. The membranes and powders with different oxidative levels were prepared using modified Hummers and Brodie synthetic procedures. A comparative study of the sorption of acetonitrile, water, pyridine, and 1-octanol was performed. It was demonstrated that dry membranes and powders, though identically synthesized and showing similar C/O ratios and inter-plane distances in the dry state, had different sorption properties at ambient temperatures. However, the difference disappeared when the temperature was lowered and did not exist for water. Water was equally sorbed into membranes and powders at ambient and lower temperatures. It was also found that the decrease in the orientational order of the membranes led to an increase in sorption. In practice, this could allow one to tune the swelling and transport properties of GO membranes directly by adjusting their internal ordering without the use of any composite materials.

From the point of view of the practical application of GO membranes for gas separation, the resistance of the internal structure of the membrane to the applied pressure is of great importance. The authors of paper [4] studied the creation of pressure-resistant GO membranes by introducing carbon-based spacers—oxidized derivatives of fullerenes C_60_(OH)_26–32_ and graphene oxide nanoribbons—into the inter-plane space. The permeability of the native and composite membranes for various gases (H_2_, He, CH_4_, N_2_, O_2_, CO_2_, C_4_H_10_, and SF_6_), as well as water vapor, was measured. The obtained results clearly demonstrate that, compare to belt-shaped nanoribbons, the ball-shaped fullerenols provide both excellent resistance towards pressure gradients and lower parasitic gas permeance. An interesting finding of the study is the fact that, despite the larger inter-plane distance and higher water sorption capacity of composite membranes, they show no enhancements in the transport of water vapors. On the contrary, a ~20–30% decrease in absolute water–vapor permeance was observed. This observation was attributed to the jamming of excessive diffusion paths for water molecules inside the graphene oxide galleries.

A serious problem when using GO membranes to separate hydrated ions is the instability of the membranes in water. To improve stability, metal cations are often introduced to the membrane structure to promote crosslinking between individual GO planes. In paper [5], GO membranes were modified with Al^3+^ from three different sources: alumina, aluminum chloride, and aluminum foil. It was showed that the membranes functionalized with Al^3+^ from aluminum foil were the most stable in water under mechanical stress; hence, the source of the modifier matters greatly. The authors attribute the observed effect to the reactivity of each Al species in acidic solution, which affected the solution pH and the number of carboxylate groups available for intra-layer crosslinking. The findings can be used in development of simple strategies for precise engineering of the GO membranes properties, which engineering is based on the conditions used in the membrane assembly process.

Paper [6] was devoted to the possibility of using the sorption properties of oxidized graphene flakes with respect to uranium in order to accurately quantify its concentration in water. The authors developed a method based on X-ray fluorescence for the facile screening of uranium in brackish water samples. Graphene oxide nanosheets were added to uncontaminated brackish water sampled from different sites to adsorb any uranium present in the samples. The nanosheets were then collected using a membrane filter and analyzed using XRF. The results revealed that the signal intensity of the U Lα peak was proportional to the water salinity. Hence, in the event of uranium release into the environment due to an accident, the content of uranium in water must be compared with the natural content of this element in water of a given salinity, and thus an excessively high concentration of uranium can be detected.
